# Reviewing the journey to the clinical application of bacteriophages to treat multi-drug-resistant bacteria

**DOI:** 10.1186/s12879-023-08621-1

**Published:** 2023-10-03

**Authors:** Gerald Mboowa

**Affiliations:** 1 African Centre of Excellence in Bioinformatics and Data-Intensive Sciences, the Infectious Diseases Institute, College of Health Sciences, Makerere University, P.O Box 22418, Kampala, Uganda; 2 Africa Centres for Disease Control and Prevention, African Union Commission, Roosevelt Street, P.O. Box 3243, Addis Ababa, W21 K19 Ethiopia

## Abstract

Antimicrobial resistance (AMR) was a leading cause of death globally in 2019. Sadly, COVID-19 has exacerbated AMR, nonetheless, the process of developing new antibiotics remains very challenging. This urgently requires the adoption of alternative approaches to treat multi-drug-resistant bacterial infections. This editorial introduces the ‘Bacteriophages against multi-drug resistant bacteria’ collection launched at BMC Infectious Diseases which highlights progress towards using bacteriophages to tackle AMR.

## Introduction

The World Health Organization (WHO) has indicated that the global new antibiotic discovery pipeline is inadequate to address the mounting threat of antimicrobial resistance (AMR) [1] which is projected to cause about 10 million deaths annually by 2050. As part of the response, the WHO generated a list of priority pathogens to guide and promote research and development (R & D) of new antibiotics, in part to address the growing global resistance to antimicrobial medicines. This denotes that multi-drug-resistant (MDR) bacteria remain one of the most important threats to global public health and are usually caused by excessive antimicrobial usage, inappropriate use of antimicrobials, and sub-standard pharmaceuticals. One of the most promising alternative strategies to treat MDR bacterial infections is utilizing bacteriophages, which constitute unique viruses that are known to lyse and kill specific bacteria. The critical entry point for phage therapy into the clinical application has been proposed to include their potential use to augment available antibiotics’ effectiveness while at the same time safeguarding newly developed drug formulations and providing a last-resort therapy in response to complete clinical antibiotic failure [2]. This phenomenological phage research has already provided efficacious clinical outcomes [3] during approved compassionate use and clinical trials in many high-income economies.

Since 2018, research demonstrating the application of bacteriophages against MDR bacterial infections has been steadily increasing. This has been matched by the global bacteriophage market that is projected to reach a value of USD 1,441.3 million by 2028 [4]. The increasing emergency of infections caused by MDR bacteria and the decline in novel antibiotic discovery has led to significant progress in the experimentation of different phages to treat drug-resistant infections [5], therefore making this a critical innovation in the management of complicated cases of drug-resistant bacterial infections. There are also growing reports regarding the simultaneous application of phages and antibiotics in patients presenting with severe MDR bacterial infections.

A group of bacterial pathogens abbreviated as ESKAPE standing for; *Enterococcus faecium*, *Staphylococcus aureus*, *Klebsiella pneumoniae*, *Acinetobacter baumannii*, *Pseudomonas aeruginosa*, and *Enterobacter* species are the leading cause of most MDR hospital-acquired infections (nosocomial) throughout the world causing life-threatening infections amongst the critically ill and immunocompromised patients. The development of novel MDR alternative treatment approaches such as the application of phage therapy has mainly targeted these pathogens. This is partly because besides causing serious nosocomial infections, ESKAPE organisms utilize a diverse array of antibiotic resistance and virulence mechanisms with the potential to disseminate them to other clinically important bacteria. Both MDR *Klebsiella pneumoniae* and *Acinetobacter baumannii* have been listed on the WHO’s most critical group of pathogens for prioritization regarding R & D of new antibiotics or other alternative therapies. These bacteria have become resistant to a large number of commonly used antibiotics, including third-generation cephalosporins and carbapenems. The latter reduces both the available treatment options and survival rates of the patients.

Some of the selected recent phage therapy reports, for example, have isolated a lytic phage against extensively drug-resistant *Acinetobacter baumannii*, a common opportunistic nosocomial pathogen that is responsible for MDR infections [6] including infections in the blood, urinary tract, pneumonia, or in wounds in other parts of the body. A second report has demonstrated a unique application of phage–antibiotic synergy in a successful combination of pre-adapted bacteriophage therapy and antibiotics for the treatment of fracture-related infection due to pan-drug-resistant *K. pneumoniae* [7]. *K. pneumonia*e is a member of the family Enterobacteriaceae and most often found associated with infections in healthcare settings and infections may be endogenous such as pneumonia, bloodstream infections, meningitis, and urinary tract infections. Compassionate use of bacteriophages for severe persistent infections during the first 5 years of the Israeli Phage Therapy Center showed that their number of registered requests was growing annually with MDR bacteria accounting for 38% of all phage requests [8].

Globally in 2021, at least 137 phage targets were listed from 135 academic and commercial phage facilities and 92 organizations such as phage banks, and biotechnology companies [9], all involved in phage-related R & D. The path from experimentation to widespread clinical use is faced with a number of challenges that need to be adequately addressed. These include, but not limited to treatment algorithm, standardization of treatment protocols for accurate therapeutic dosing, storage conditions or stewardship, duration of use, and patient monitoring, as well as the development of consensus on a standard set of outcome measures to demonstrate successful clinical utility [10]. The antibacterial range of phages is typically much lower than that of most antibiotics, but this potentially decreases the risk of phage therapy on the natural microflora composition of the human body like in the gastrointestinal tract, unlike commonly used antibiotics. The clinical safety of the application of therapeutic phages compared to other options may also be considered a significant benefit [3] in the post-antibiotic era. Since MDR bacterial infections arise largely due to the overuse of antibiotics, phage therapy will greatly reduce the emergence of antibiotic resistance in bacteria when used to complement the available treatment options. This situation stands to safeguard current precious last-resort antibiotics by immensely lessening the drug pressure exerted on bacteria and prolonging the number of years needed for these antibiotics to remain effective.

As a Guest Editor of this Collection titled, “Bacteriophages against multi-drug resistant bacteria”, I am honored to invite infectious diseases experts to contribute high-quality original research articles on advances made in the use of bacteriophages as a treatment for multi-drug and pan-drug resistant bacteria. This may include efficacy data generated through clinical trials or even compassionate phage therapy. Furthermore, manuscripts may also include work that explores the mechanisms of phage-bacteria interactions, the impact of phage therapy on the natural microflora composition of the human body, the development and optimization of phage therapy protocols, and findings from applications of phage therapy in tissues, humans, and animals among others. I am motivated to believe that this collection will greatly contribute to an in-depth understanding of this well-timed promising field and facilitate further innovation to guide the scale-up of phage therapy.

## Data Availability

Not applicable.

## Abbreviations

AMR: Antimicrobial resistance
MDR: Multidrug-resistant
R & D: Research and Development
